# A Dual-Color Bioluminescence Reporter Mouse for Simultaneous *in vivo* Imaging of T Cell Localization and Function

**DOI:** 10.3389/fimmu.2018.03097

**Published:** 2019-01-08

**Authors:** Jan Willem Kleinovink, Laura Mezzanotte, Giorgia Zambito, Marieke F. Fransen, Luis J. Cruz, J. Sjef Verbeek, Alan Chan, Ferry Ossendorp, Clemens Löwik

**Affiliations:** ^1^Department of Immunohematology and Blood Transfusion, Tumor Immunology, Leiden University Medical Center, Leiden, Netherlands; ^2^Department of Radiology and Nuclear Medicine, Optical Molecular Imaging, Erasmus Medical Center, Rotterdam, Netherlands; ^3^Department of Molecular Genetics, Erasmus Medical Center Rotterdam, Netherlands; ^4^Medres, Cologne, Germany; ^5^Translational Nanobiomaterials and Imaging, Department of Radiology, Leiden University Medical Center, Leiden, Netherlands; ^6^Department of Human Genetics, Leiden University Medical Center, Leiden, Netherlands; ^7^Percuros B.V., Enschede, Netherlands; ^8^Department of Oncology, CHUV Lausanne University Hospital, Lausanne, Switzerland

**Keywords:** T cell, activation, mouse, dual-color, luciferase, bioluminescence, imaging, reporter

## Abstract

Non-invasive imaging technologies to visualize the location and functionality of T cells are of great value in immunology. Here, we describe the design and generation of a transgenic mouse in which all T cells constitutively express green-emitting click-beetle luciferase (CBG99) while expression of the red-emitting firefly luciferase (PpyRE9) is induced by Nuclear Factor of Activated T cells (NFAT) such as during T cell activation, which allows multicolor bioluminescence imaging of T cell location and function. This dual-luciferase mouse, which we named TbiLuc, showed high constitutive luciferase expression in lymphoid organs such as lymph nodes and the spleen. *Ex vivo* purified CD8+ and CD4+ T cells both constitutively expressed luciferase, whereas B cells showed no detectable signal. We cross-bred TbiLuc mice to T cell receptor-transgenic OT-I mice to obtain luciferase-expressing naïve CD8+ T cells with defined antigen-specificity. TbiLuc^*^OT-I T cells showed a fully antigen-specific induction of the T cell activation-dependent luciferase. In vaccinated mice, we visualized T cell localization and activation in vaccine-draining lymph nodes with high sensitivity using two distinct luciferase substrates, D-luciferin and CycLuc1, of which the latter specifically reacts with the PpyRE9 enzyme. This dual-luciferase T cell reporter mouse can be applied in many experimental models studying the location and functional state of T cells.

## Introduction

Bioluminescence imaging (BLI) is an optical molecular imaging technique based on the emission of light produced by luciferase enzymes expressed in cells or animals. It has been extensively used to study gene expression, using genetic constructs in which expression of the luciferase is driven by the promoter of the gene of interest (1). A common application of BLI is the monitoring of tumor cell growth using luciferase-expressing tumor cells (2). Besides functioning as a quantitative measure for cell number, BLI also provides information on cell viability as the light-producing reaction mediated by firefly or click beetle luciferases requires ATP, oxygen and Mg^2+^, thus, the context of a living cell (3, 4). Commonly, retroviral or lentiviral constructs harboring the luciferase gene coupled to a specific promoter are designed to integrate the luciferase gene into cells by viral transduction. Alternatively, luciferase-transgenic reporter mouse strains can be developed using similar constructs, requiring more time and effort but becoming a source of luciferase-expressing cells without the need for further modification. Such transgenic models are especially valuable if the cells of interest are to be studied in their natural, unmodified state.

BLI technology is highly suited to analyze the immune system *in vivo*, because it allows real-time visualization of the typically strong dynamics of many immune cells, which often change location and expand or contract in number over short periods of time. These characteristics are particularly true for T cells, which are found in high numbers in lymphoid organs such as the spleen and lymph nodes, but also circulate in the bloodstream to patrol the body and enter peripheral tissues in case these harbor their target. These properties make T cells an attractive target for BLI. Thus far, many attempts to create luciferase-expressing T cells have used viral transduction of T cells. However, T cells in their untouched natural state, immunologically referred to as ‘naïve T cells’, are practically impossible to transduce. Therefore, in order to allow their transduction, T cells are first activated *in vitro* to facilitate their transduction, and then rested for several days before use in an experiment (5–9). However, the transition of a T cell from the naïve to the activated state is not fully reversible, as T cell activation starts transcriptional programs that cannot be reversed. Hence, although commonly overlooked, the results obtained with BLI of such transduced T cells cannot be directly compared to the natural situation in which T cells are naïve when they first encounter their target. These drawbacks have led to the production of a number of T cell luciferase-transgenic mouse models to allow the tracking of T cells (10–12). While definitely a step forward from using transduced T cells, these single-luciferase transgenic models have the limitation that they only provide information on the location of T cells, but not their functional state.

Recently, Szyska et al. published a dual reporter mouse that ubiquitously expresses Renilla luciferase and NFAT-driven click beetle red luciferase CBRed (13). Dual-color imaging is achieved by using the substrates Coelenterazine and D-luciferin. Considering that Renilla luciferase is less bright than the green luciferase mutant CBG99 (14) and that Coelenterazine substrates give higher background than D-luciferin and show suboptimal bioavailability and stability (15, 16), we aimed to create a system that does not employ Coelenterazine but shows good sensitivity for T cell imaging, especially for longitudinal studies. We have previously shown that the click-beetle green luciferase mutant CBG99 and the red-emitting firefly mutant PpyRE9 can be efficiently combined for multicolor *in vivo* bioluminescence imaging of transplanted cells previously transduced with a single luciferase, using the substrate D-luciferin (17). In this study, we show the design and generation of a transgenic mouse model called TbiLuc, whose combination of a constitutive and an inducible luciferase in T cells allows dual-color visualization of T cell location and function. In TbiLuc, all T cells constitutively express the green CBG99 luciferase driven by the human CD2 promoter, and the transcription factor Nuclear Factor of Activated T cells (NFAT) induces the expression of the red PpyRE9 luciferase in addition.

We show that luciferase expression is restricted to T cells, and that antigen-specific or non-specific activation of T cells successfully induces the expression of the NFAT-dependent luciferase. As the expression level of the two luciferases influences the ability to efficiently separate the two light signals *in vivo* using a single substrate, we combined the recently developed luciferase substrate CycLuc1 as a specific substrate for firefly luciferases (such as PPyRE9) (18) with D-luciferin as a substrate for the CBG99 enzyme. As we show that CycLuc1 is not a functionally efficient substrate for CBG99, we could efficiently separate light signals *in vivo*. Furthermore, we cross-bred TbiLuc mice to T cell receptor-transgenic OT-I mice and subsequently localized vaccine-specific CD8^+^ T cells in lymphoid organs and visualized their activation upon vaccination. Altogether, we have developed a dual-luciferase T cell reporter mouse that allows live bioluminescence imaging of T cell location and function, which has numerous possible applications in many experimental models where T cells play a central role.

## Materials and Methods

### Mice and Cell Lines

The following construct was designed for the generation of the TbiLuc transgenic mouse. The PpyRE9 gene [Photinus pyralis red-emitting luciferase 9, a kind gift of Prof. Branchini (19)] was cloned downstream of 3 repetitive NFAT response elements and minimal promoter derived from the pGL4.30 plasmid (Promega, Madison, USA). The sequence of the human CD2 promoter was cloned upstream of the CBG99 green click beetle luciferase gene (20, 21). These two cassettes were cloned to form a bidirectional construct, separated by an insulator. TbiLuc mice were generated by injection of the bicistronic construct into pronuclei of fertilized oocytes of CBA^*^C57BL/6 mice. In the pups born the presence of the transgene was determined by a specific PCR using genomic DNA from tail biopsy and its activity/function was measured by evaluation of light emission from tail-vein blood after addition of the luciferase substrate D-luciferin. Mice were back-crossed to the C57BL/6 strain for several generations before use in experiments. Albino B6 mice (tyrosinase-deficient immunocompetent C57BL/6 mice), TbiLuc mice (dual T cell luciferase transgenic mice), OT-I mice (T cell receptor-transgenic mice carrying the CD45.1 congenic marker) and crossed TbiLuc^*^OT-I mice were bred in the animal breeding facility of the Leiden University Medical Center, the Netherlands. All experiments were approved by the animal ethical committee of Leiden University. D1 is a GM-CSF-dependent immature dendritic cell line derived from C57BL/6 mice, and B3Z is an OVA-specific CD8 T cell hybridoma carrying the lacZ reporter gene induced by NFAT (22, 23). Cell lines were assured to be free of rodent viruses and *Mycoplasma* by regular PCR analysis. Cells were cultured as previously described (24).

### Bioluminescence Imaging (BLI)

#### In Vitro

Cell samples were prepared for *in vitro* BLI analysis in sterile black-walled flat-bottom 96-wells plates (Greiner, Alphen aan den Rijn, The Netherlands). Cells were suspended in 100 μL PBS containing 1 mM D-luciferin potassium salt (SynChem, Felsberg, Germany) or 0.1 mM CycLuc1 (Aobious, Gloucester, MA, USA), incubated for 5 min at 37°C. BLI imaging was performed using an IVIS Spectrum small animal imager (PerkinElmer, Waltham, MA) that measured the light signal using open filter and a series of 20 nm wavelength band filters from 500 to 700 nm, with an acquisition time of 30 s. Accompanying LivingImage 4.2 software (Perkin Elmer) was used for spectral unmixing of the full-spectrum measurement to identify individual signals *in vitro*.

#### Ex Vivo

For characterization of the TbiLuc model, a group of 3 TbiLuc mice were injected intraperitoneally with 150 mg/kg D-luciferin, anesthetized by isoflurane inhalation and imaged after 10 min (peak of emission) using an IVIS Spectrum imager set at the “open” filter with an exposure time of 60 s. Another group of 3 TbiLuc mice were injected intraperitoneally with 7,6 mg/kg CycLuc1, left for 5 min and anesthetized by isoflurane inhalation for IVIS imaging. Next, organs from TbiLuc mice were taken out and rinsed in PBS before BLI analysis, using an acquisition time of 30 s. Signal quantification in specific regions of interest (ROIs) was performed depicting regions of interest (ROIs) corresponding to the whole organ and signals were corrected for background by subtracting the signal from the same size of ROIs placed at irrelevant positions. Data are reported as photons flux per mg of tissue.

#### In Vivo

Mice (*n* = 8) were injected with 150 mg/kg D-luciferin, anesthetized by isoflurane inhalation and imaged after 10 min (peak of emission) using the “open” filter and 560 nm filter with an exposure time of 30 seconds. After 3 h, mice were imaged to assure that no D-luciferin-mediated signal was present anymore, and mice were subsequently injected intraperitoneally with 7,6 mg/kg CycLuc1, left for 5 min and anesthetized by isoflurane inhalation. This concentration of CycLuc1 was chosen as a maximum injectable dose given its low solubility. Mice were imaged using open filter and 620 nm filter with an exposure time of 30 s. Signal quantification in specific regions of interest (ROIs) was performed by using fixed-size and fixed-position ROIs throughout the experiments, and signals were corrected for background by subtracting the signal from ROIs placed at irrelevant positions.

### Isolation of Immune Cells

Immune cells were obtained from the spleen by mashing on 70 μm cell strainers (BD Biosciences, San Jose, CA, USA) to create single-cell suspensions, followed by erythrocyte lysis. Then, CD4^+^ and CD8^+^ T cells were isolated separately by negative magnetic selection (BD IMag enrichment kits), B cells by positive magnetic selection of B220^+^ cells (BD IMag), while NK cells and myeloid cells were obtained by FACS-sorting for CD3^−^/NK1.1^+^/NKp46^+^ (NK cells) and CD3^−^/CD11b^+^ (myeloid cells).

### T Cell Activation *in vitro*

T cells isolated from spleens of TbiLuc or TbiLuc^*^OT-I mice were stimulated overnight with 100 ng/mL Phorbol 12-myristate 13-acetate (PMA) + 1500 ng/mL ionomycin (both Sigma-Aldrich, St. Louis, MO, USA), with agonistic anti-CD3 and anti-CD28 antibodies (BD Biosciences) pre-coated at 1 μg/mL (unless indicated otherwise) at 37°C for 30 min, or with 50,000 D1 dendritic cells pre-loaded with OVA immune complexes. OVA immune complexes were formed by incubating a 1:300 mass ratio of OVA protein (Worthington, Lakewood, NJ, USA) and anti-OVA antibody (LSBio, Seattle, WA, USA) for 30 min at 37°C, after which the immune complexes were added to D1 dendritic cells and incubated overnight. Unloaded D1 cells were incubated overnight in parallel to serve as control cells.

### Adoptive Transfer and Vaccination

Adoptive transfer consisted of 1 million purified CD8^+^ OT-I cells (unless stated otherwise) isolated as described above, injected intravenously in 200 μL PBS in the tail vein. Vaccination consisted of subcutaneous injection of 1 million (unless indicated otherwise) D1 cells pre-loaded with OVA immune complexes, or unloaded control D1 cells, in 50 μL PBS in the tail-base region.

### Western Blot

Expression of PpyRE9 luciferase by activated T cells was confirmed by Western Blot. T cells were isolated and stimulated overnight as described above. Then, 3^*^10^6^ cells were lysed and the total protein content of each sample was determined by a Pierce BCA protein assay kit (Thermo Scientific, Rockford, USA). Next, 20 μg of total cell extract was applied to a 10% SDS-PAGE and transferred onto a nitrocellulose membrane. After washing, the membrane was incubated overnight with rabbit anti-luciferase polyclonal antibody in TPBS at 1/500 dilution (Fitzgerald, Acton, USA). The GAPDH antibody (Cell Signaling Technology, Danvers, MA, USA) was used to correct for the amount of total protein. The blots were washed, exposed to an HRP-conjugated secondary antibody for 1 h, and detected using enhanced chemiluminescence (ECL) reagents (Thermo Scientific). Detection of ECL signals was performed with the IVIS Spectrum and quantification of bands using Living Image Software 4.2.

### Flow Cytometry

Before adoptive transfer of CD8 T cells, their CD8-purity and naïve phenotype was assessed by flow cytometry. In short, spleen cells were suspended in FACS buffer (PBS with 0.5% FCS and 0.02% sodium azide), surface-stained with antibodies against CD8β, CD44, CD45.1, and CD62L (BioLegend, San Diego, CA, USA) and analyzed on a BD Accuri C6 flow cytometer. Analysis was performed on FlowJo software (FlowJo, USA).

### Statistical Analysis

Statistical analysis was performed using GraphPad Prism 6.0 software. Data are shown as the mean ± SEM for each group, and comparison of groups was performed by two-tailed Student's *t*-test. Statistical differences were considered significant at *p* < 0.05.

## Results

### Design and Development of the Dual-Luciferase Transgenic TbiLuc Mouse

**Figure 1:**
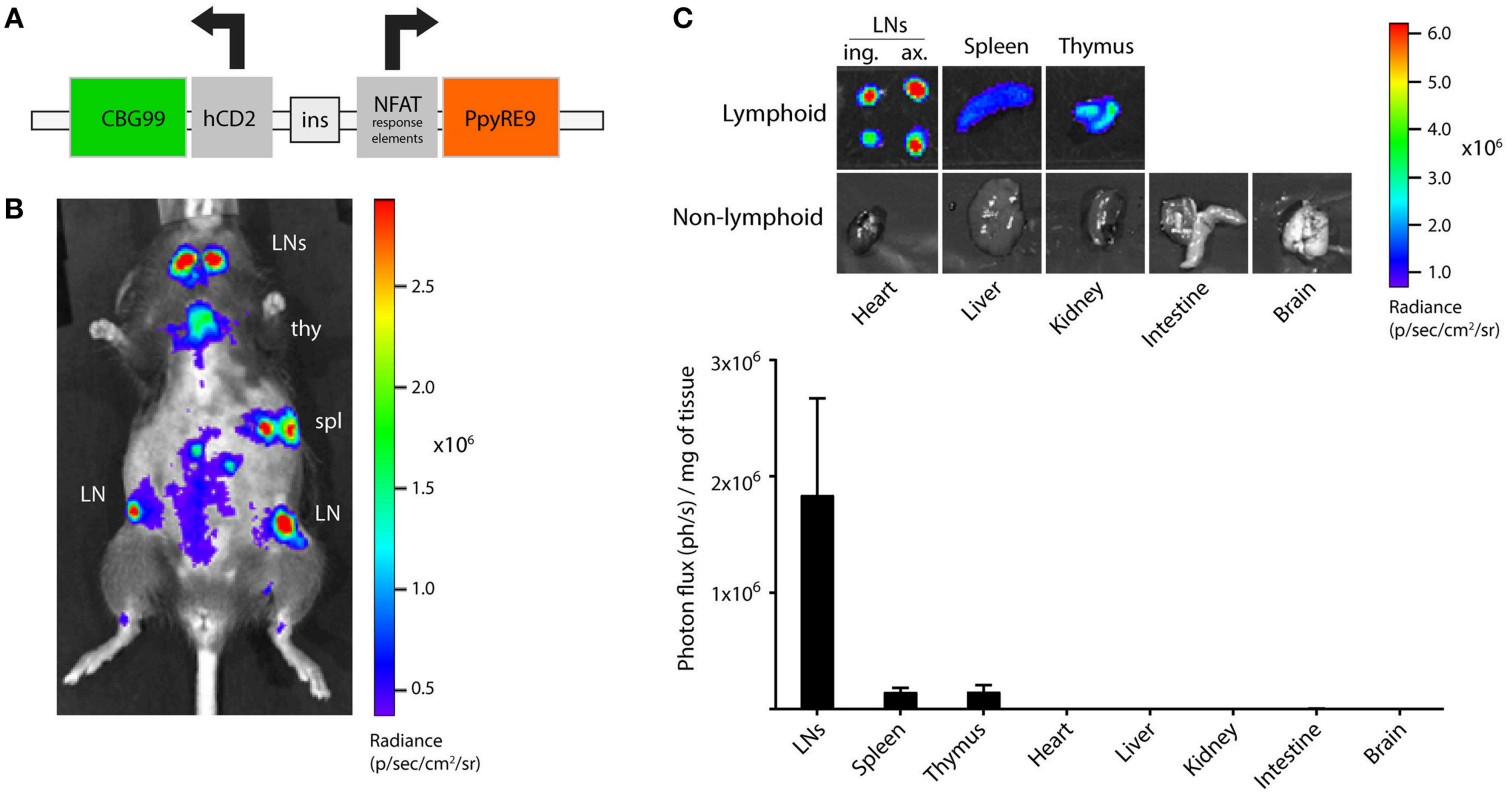
Design and development of the dual-luciferase transgenic TbiLuc mouse. **(A)** Schematic overview of the dual-luciferase reporter bicistronic vector. NFAT, nuclear factor of activated T cells; CBG99, click beetle green luciferase 99; PpyRE9, *Photinus pyralis* red-emitting luciferase 9. **(B)** Bioluminescence measurement of a TbiLuc mouse using the D-luciferin substrate, showing strong constitutive CBG99 luciferase expression in lymphoid organs. Lymphoid organs are indicated: LN, lymph node; spl, spleen; thy, thymus. The abdomen is shaved to reduce signal absorption. **(C)** Representative images and quantification of luciferase signal in several lymphoid and non-lymphoid organs of TbiLuc mice (*n* = 3, mean + SEM). LNs, lymph nodes; ing., inguinal; ax., axillary.

We designed a bicistronic vector containing the click beetle green luciferase (CBG99) sequence under the control of the human CD2 (hCD2) promoter and the red-emitting firefly luciferase (PpyRE9) sequence under the control of the nuclear factor of activated T cells (NFAT) minimal promoter (Figure 1A). Background NFAT expression in naïve T cells is low and increases strongly after T cell activation, making it an ideal promoter to visualize activated T cells. The hCD2 promoter is expressed in both T cells and B cells in humans, but is T cell-specific when used in mice (20). This bicistronic vector was used to generate the TbiLuc transgenic mouse on a B6/CBA mixed background. Founders were selected based on high light emission in blood and further back-crossed more than 10 times toward B6 background. The CD2-driven CBG99 luciferase was constitutively expressed in lymphoid organs, which was evident both in live *in vivo* BLI and upon dissecting lymphoid and non-lymphoid organs showing on average a 1,000-fold higher signal in lymphoid organs than in non-lymphoid organs (Figures 1B,C).

### Luciferase Expression Is Restricted to T Cells

**Figure 2:**
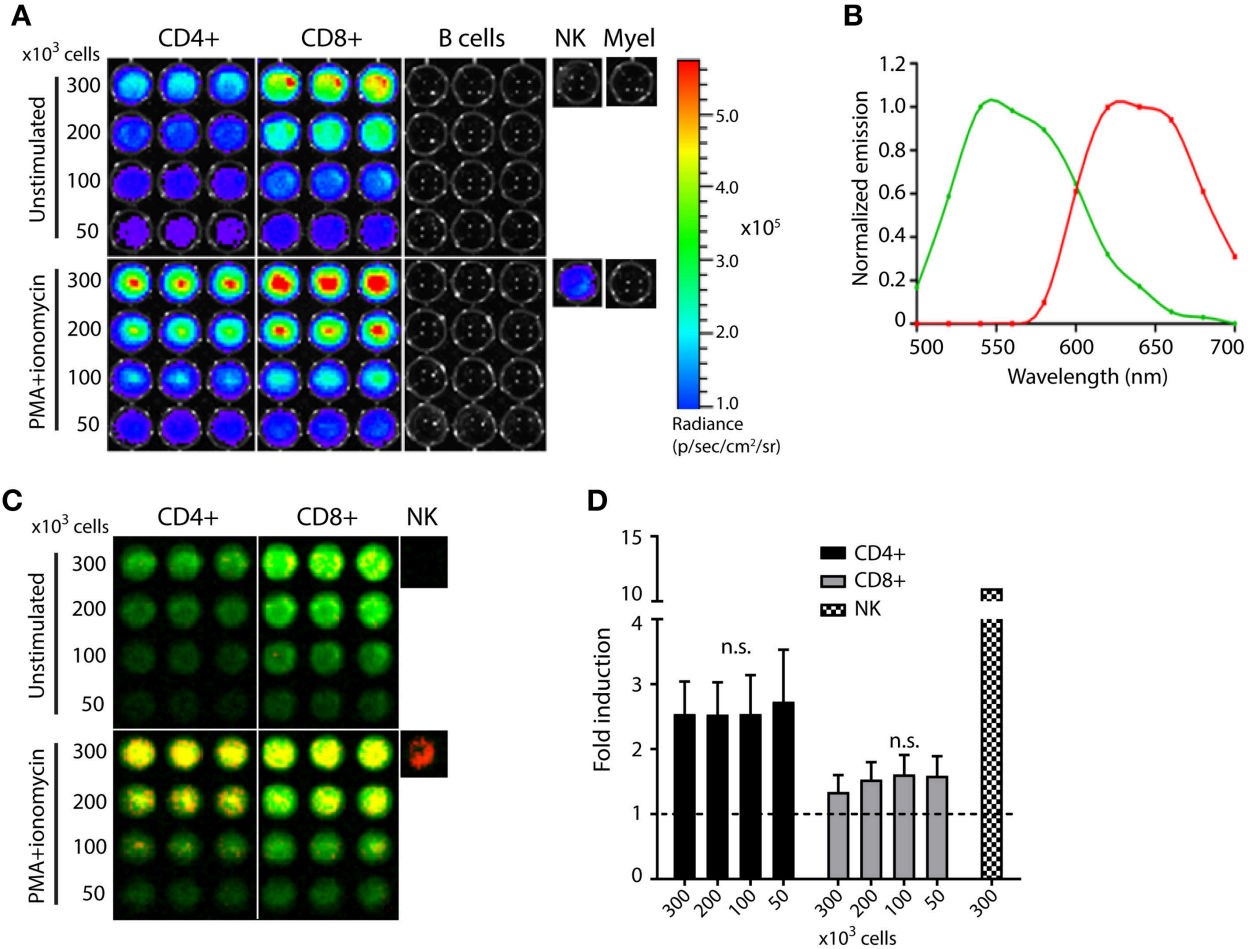
T cell-specific expression of constitutive and inducible luciferases in the TbiLuc mouse. T cells, B cells, NK cells, and myeloid cells were isolated from the spleen of TbiLuc mice, plated in different numbers, and stimulated overnight with PMA + ionomycin to activate the NFAT-inducible red luciferase. Representative data from two independent experiments. **(A)** Luciferase expression is restricted to CD4 and CD8 T cells and signal strength correlates to the number of T cells, both in unstimulated (top half) and PMA+ionomycin stimulated cells (bottom half). **(B)** Spectral unmixing of the total BLI signal identifies two emission spectra matching the constitutive CBG99 luciferase (green) and the NFAT-inducible PpyRE9 luciferase (red). **(C)** Spectral unmixing applied to the signals from Figure A indicates that naïve T cells express only constitutive CBG99 luciferase (top half, green color), while activated T cells express both luciferases (bottom half, overlay of green and red signals yielding yellow). NK cells produce PpyRE9 after stimulation but do not express CBG99 luciferase. **(D)** Fold induction of NFAT-luciferase expression in TbiLuc cells after PMA+ionomycin stimulation compared to unstimulated cells. Signals are corrected for differences in constitutive hCD2-luciferase expression. Baseline expression (1-fold) is indicated by the dotted line. Representative data of two independent experiments with *n* = 3 samples per group each (*n* = 1 for NK cells). Mean + SD, the fold induction within a cell subset is independent of cell number as there are no significant differences within CD4 T cells or within CD8 T cells (one-way ANOVA).

Next, we analyzed in more detail which cells in the lymphoid organs express luciferase by isolating CD4 T cells, CD8 T cells, B cells, NK cells and myeloid cells from the spleens of TbiLuc mice. Constitutive expression of CBG99 luciferase in unstimulated cells was completely restricted to T cells, as the other cell types showed no detectable bioluminescence signal (Figure 2A, top half). CD8 T cells produced a two-fold higher luciferase activity than CD4 T cells. The luciferase activity was proportional to the number of T cells. In parallel, we tested the validity of the dual-luciferase construct by stimulating these isolated immune cells with PMA and ionomycin, which are chemical compounds often used in combination to trigger NFAT by activating the protein kinase C (PKC) pathway and increasing intracellular levels of calcium, respectively. Also after PMA/ionomycin treatment, strong luciferase activity was observed only in T cells, except for a weakly detectable signal in NK cells (Figure 2A, bottom half).

### T Cell Activation Results in NFAT-Induced Luciferase Expression

We next analyzed if the increased light signal after PMA/ionomycin treatment was mediated by the NFAT-driven PpyRE9 luciferase. A spectral unmixing algorithm was applied to separate the independent emission spectra within the sample and quantify these spectra separately. Two distinct emission patterns were identified corresponding to the emission spectra of the green CBG99 and the red PpyRE9 luciferases (Figure 2B). By representing the unmixed signals in artificial green and red colors, the emission spectra of the two luciferases can be assessed per single sample. Unstimulated naïve T cells expressed only the constitutive hCD2-driven CBG99 luciferase, while PMA/ionomycin treatment induced the expression of NFAT-driven red PpyRE9 luciferase, resulting in a yellow color based on overlaying green and red colors (Figure 2C). The low signal observed in NK cells after PMA/ionomycin treatment was confirmed to be NFAT-induced, as shown by the red color. The induction of PpyRE9 luciferase expression by PMA/ionomycin treatment was further analyzed by Western Blotting, showing a clear presence of PpyRE9 luciferase protein band 24 h after stimulation of TbiLuc CD8^+^ T cells with PMA/ionomycin (Supplementary Figure 1a). We calculated the fold induction of the NFAT-luciferase after unmixing of signals and correction of red PpyRE9 signals (representing NFAT-driven expression) for the green CBG99 signals (representing constitutive T cell expression). For CD4+ T cells we found an average of 2.5- ± 0.5 -fold induction, for CD8+ T cells an average of 1.5- ± 0.1 -fold induction and for NK cells we calculated an average fold induction of 11 (Figure 2D).

### Dual-Luciferase Imaging of Antigen-Specific T Cells

**Figure 3:**
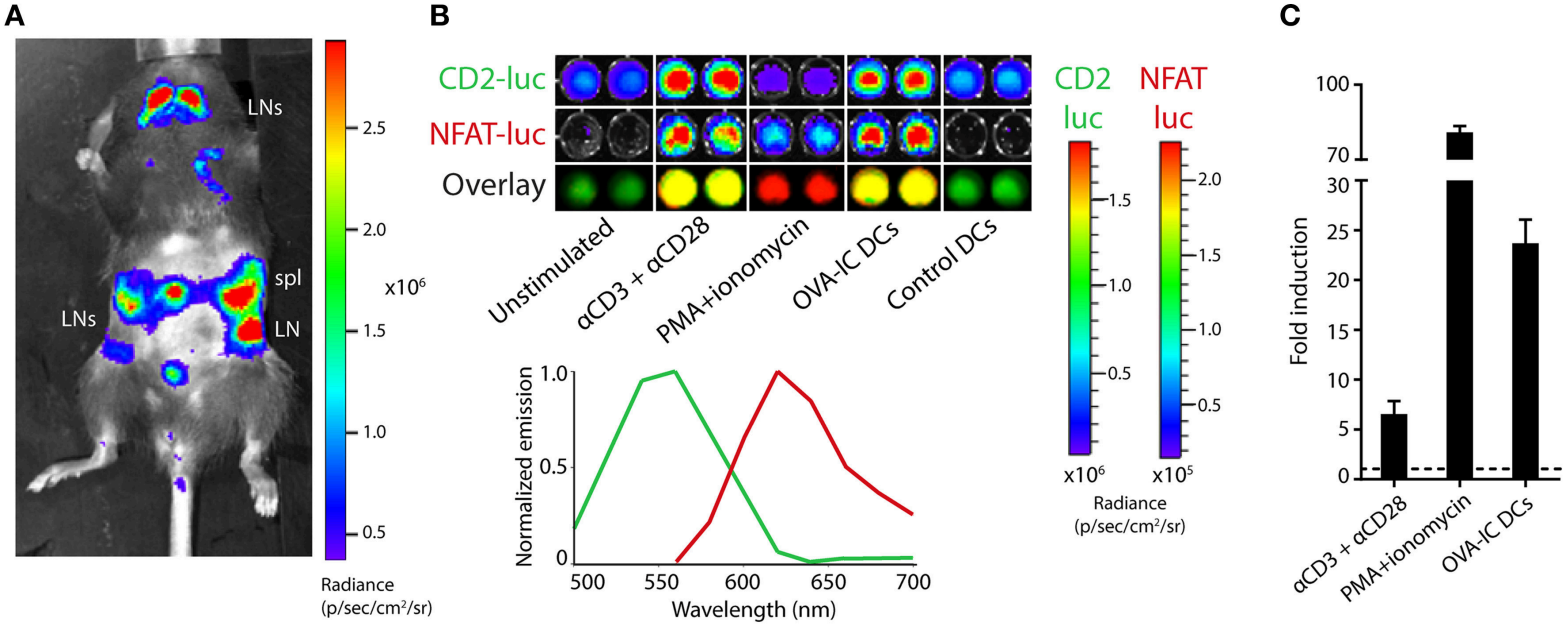
Dual-luciferase imaging of antigen-specific T cells. **(A)** Bioluminescence measurement of a TbiLuc^*^OT-I mouse, showing luciferase expression in secondary lymphoid organs. The abdomen is shaved to reduce signal absorption. The spleen (spl) and various lymph nodes (LN) are indicated. **(B)** CD8 T cells were isolated from the spleen of TbiLuc^*^OT-I mice and stimulated overnight with agonistic CD3/CD28 antibodies, PMA + ionomycin, dendritic cells loaded with OVA protein immune complexes (OVA-IC DCs) or with unloaded control DCs, as indicated. Subsequent bioluminescence imaging shows constitutive CBG99 expression (CD2-luc, top), activation-induced PpyRE9 expression (NFAT-luc, middle) and an overlay of artificial green and red colors (bottom) corresponding to the spectral unmixing as shown in the bottom-right graph. Representative data from two independent experiments. **(C)** Fold induction of NFAT-luciferase expression in TbiLuc^*^OT-I cells after stimulation with PMA+ionomycin or αCD3+αCD28 compared to unstimulated cells, or after incubation with OVA-IC DCs compared to control DCs. Signals are corrected for differences in constitutive hCD2-luciferase expression. Baseline expression (1-fold) is indicated by the dotted line. Representative data of two independent experiments with *n* = 2 samples per group each. Mean + SD.

In order to study dual-bioluminescent T cells with known antigen specificity, the TbiLuc mouse was crossed to T cell receptor-transgenic OT-I mice whose CD8 T cells recognize the SIINFEKL epitope of chicken ovalbumin (OVA) as presented in H-2K^b^ MHC class I molecules. The resulting TbiLuc^*^OT-I mice showed constitutive luciferase expression in lymphoid organs, as was the case in the parental TbiLuc mice (Figure 3A). To study NFAT-luciferase induction in an antigen-specific manner, we purified CD8^+^ T cells from the spleens of TbiLuc^*^OT-I mice by magnetic selection. This procedure yielded >90% pure CD8 T cells with a naïve phenotype (Supplementary Figure 1b). The isolated CD8 T cells were then co-incubated *in vitro* with dendritic cells presenting the SIINFEKL epitope processed from the OVA protein after uptake of immune-complexes (OVA-IC), which we have previously reported as an efficient CD8 T cell vaccine (25). This led to a strong induction of NFAT-luciferase, which was completely antigen-specific as control dendritic cells did not induce any expression, identical to unstimulated naïve T cells (Figure 3B). We calculated the fold induction of the NFAT-luciferase after unmixing of signals. For CD8+ T cells we found an average of 6.5 ± 1.3 -fold induction after T cell activation using αCD3/αCD28 antibodies, an average of 81 ± 2 -fold induction when using PMA/Ionomycin and an average of 24 ± 2 -fold induction when using OVA-IC DCs. (Figure 3C). Western Blot analysis confirmed the presence of PpyRE9 protein (Supplementary Figure 1c). Although the use of a single D-luciferin substrate was sufficient for dual-color imaging *in vitro*, the relatively weak NFAT-induced red luciferase signal does not allow efficient *in vivo* detection of T cell activation. Therefore, we employed the new luciferin substrate CycLuc1, which has specifically been designed to improve light emission from firefly luciferases such as our NFAT-induced PpyRE9 red luciferase (18). *In vivo* imaging of naïve TbiLuc mice using the CycLuc1 substrate (Supplementary Figure 2a) shows a low constitutive CBG99 luciferase signal compared to the use of the D-luciferin substrate as in Figure 1. Although BLI signals in the lymphoid organs were detectable using CycLuc1, these were 10-fold lower in lymph nodes, 3-fold lower in the spleen and 5-fold lower in the thymus when compared to BLI signals using D-luc, while BLI signals were nearly absent in non-lymphoid organs (Supplementary Figure 2b).

**Figure 4:**
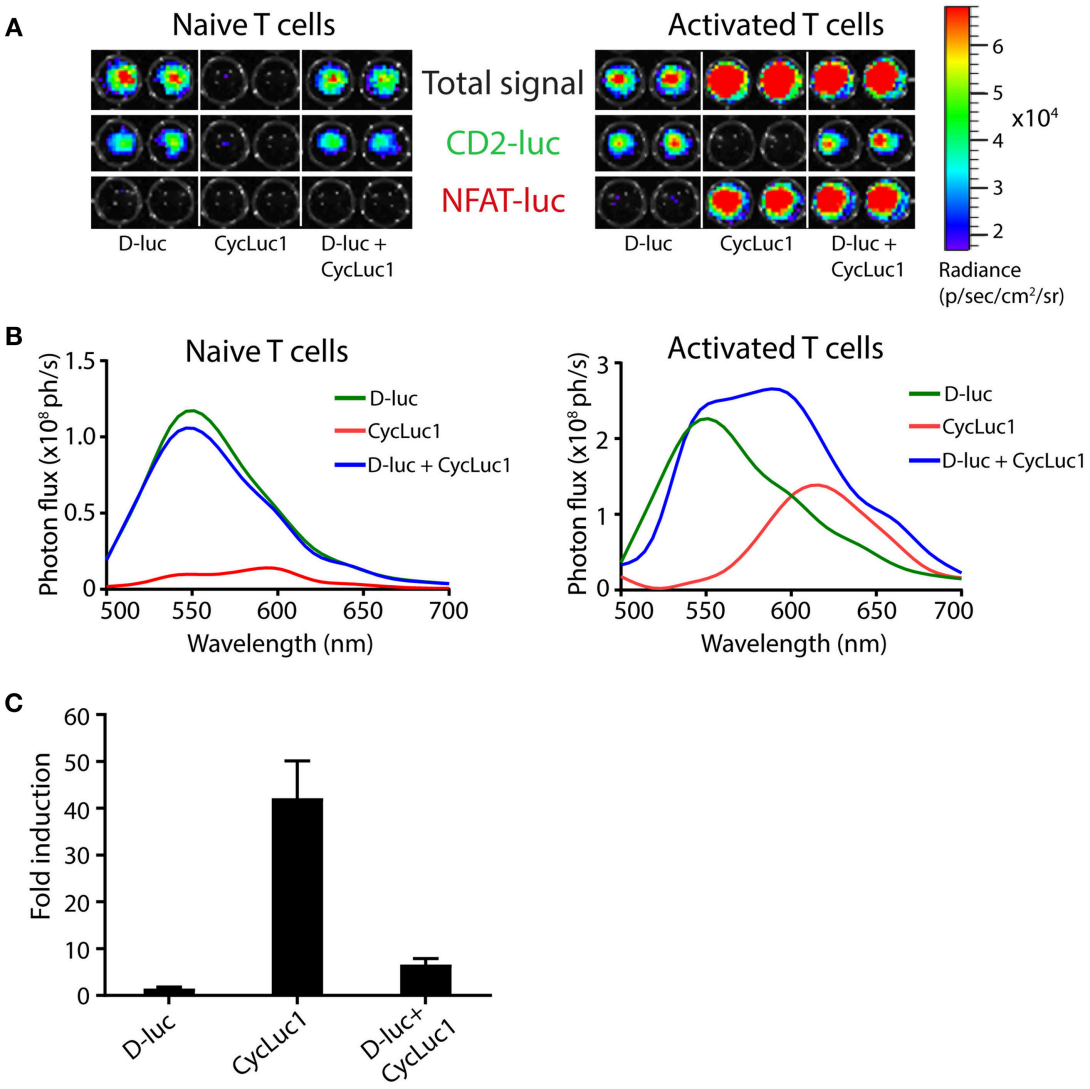
Efficient visualization of NFAT-induced PpyRE9 luciferase using the CycLuc1 substrate. **(A)** CD8 T cells from TbiLuc^*^OT-I mice left untreated (“Naive T cells”) or stimulated overnight with OVA immune complex-loaded dendritic cells (“Activated T cells”) were imaged using the substrate D-luciferin (D-luc), CycLuc1, or both, as indicated. Total signal (top), the constitutive green CD2-luciferase (middle) and the activation-induced red NFAT-luciferase (bottom) are shown separately, all on the same scale. **(B)** Emission spectra of naive T cells and of activated T cells (from Figure A) using different substrates, showing strong constitutive CBG99 signals using the D-luciferin substrate, and specific activation-induced PpyRE9 signals using CycLuc1. **(C)** Fold induction of NFAT-luciferase expression in TbiLuc^*^OT-I cells after incubation with OVA-IC DCs compared to unstimulated cells and BLI measurement using D-luc, CycLuc1 or both. Signals are corrected for differences in constitutive CD2-luciferase expression. Mean + SD. Representative data from two independent experiments.

By using T cell hybridomas expressing either one of the two luciferases also used in TbiLuc, we show that CycLuc1 is indeed an inefficient substrate for CBG99 green luciferase, as indicated by the 30-fold lower signal at the emission peak as compared to D-luciferin (Supplementary Figure 3a). This allows specific detection of PpyRE9 luciferase in TbiLuc T cells using the CycLuc1 substrate. Moreover, these data indicate that substrates given in combination compete for the active site of the enzymes, as shown by the 1,6 times lower average photon flux measured for CBG99 emission with D-Luc + CycLuc1 as compared to D-Luc alone. Next, we investigated whether dual-color imaging of TbiLuc^*^OT-I T cell activation could be performed using the two substrates, D-luciferin and CycLuc1. Addition of a single substrate per sample allowed efficient detection of the constitutive signal from CBG99 green luciferase by D-luciferin, and the activation-induced signal from PpyRE9 red luciferase by CycLuc1 (Figure 4A). Unlike naïve T cells, activated T cells expressing both luciferases produce a spectrum with no isolated peaks of emission when the two substrates are added simultaneously (Figure 4B). For CD8+ T cells we found an average of 1.5 ± 0.1 -fold induction of NFAT-luciferase expression using D-luciferin and an average of 42 ± 8 -fold induction using CycLuc1 and an average of 7 ± 1 -fold induction using combination of substrates (Figure 4C). Therefore, separate addition of substrates is warranted for efficient separation of light signals in activated T cells. We estimated the sensitivity of detecting T cell activation with CycLuc1 *in vitro* by showing that stimulation with agonistic anti-CD3 antibodies up to 100x below the optimal concentration still results in NFAT-luciferase signals detectable by CycLuc1 (Supplementary Figure 3b). In addition, the use of the CycLuc1 substrate for visualization of activation-induced PpyRE9 luciferase was also validated in CD4^+^ and CD8^+^ T cells from parental TbiLuc mice (Supplementary Figure 3c).

### Visualization of T Cell Localization and Activation in Vaccinated Mice

**Figure 5:**
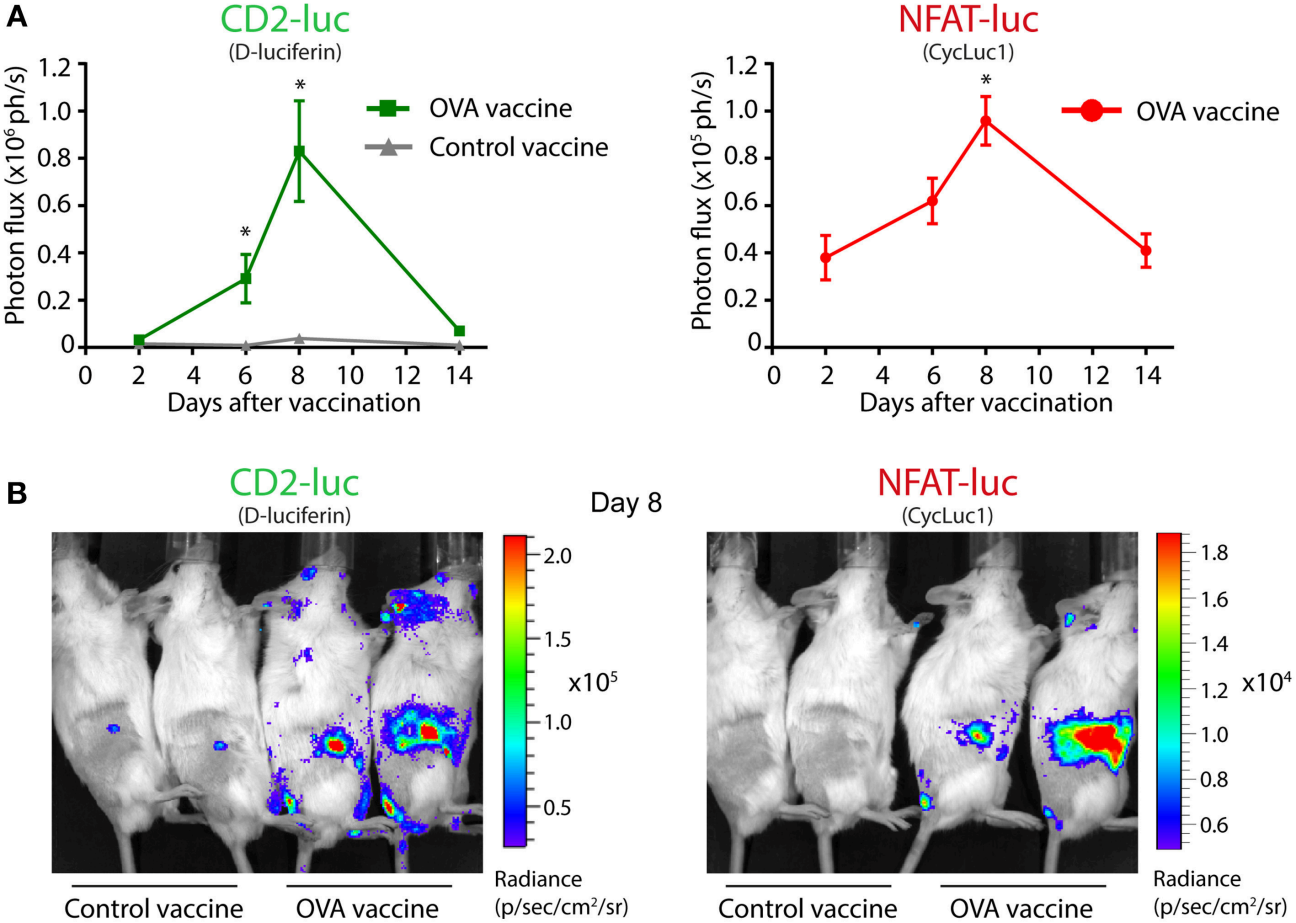
Visualization of T cell localization and activation in vaccinated mice. **(A)** Constitutive CD2-luciferase (left) and NFAT-luciferase (right) signals from vaccine-draining inguinal lymph nodes of vaccinated mice. On day 0, mice received adoptive transfer of TbiLuc^*^OT-I CD8+ T cells and were vaccinated with OVA immune complex-preloaded dendritic cells or with unloaded control dendritic cells. Two representative mice are shown per group from a total of 4 mice per group. CD2-CBG99 and NFAT-PpyRE9 luciferases were visualized using the D-luciferin and CycLuc1 substrates, respectively. Mice receiving the control vaccine showed no detectable NFAT-luc signal after subtraction of background. Statistical significance of differences in signal compared to day 2 is indicated by asterisks, ^*^p < 0.05. **(B)** Representative pictures of OVA-vaccinated and control-vaccinated mice on day 8, showing dramatic T cell proliferation (left) and activation (right) in OVA-vaccinated mice.

We set out to investigate whether antigen-specific target recognition by T cells could be visualized *in vivo*, using an adoptive T cell transfer system. First, we visualized the fate of T cells after adoptive transfer. Intravenously injected TbiLuc^*^OT-I CD8^+^ T cells had efficiently homed to the lymphoid organs of recipient Albino B6 mice after 1 day, and the number of transferred T cells correlated to the signal strength (Supplementary Figure 4). Next, mice were vaccinated subcutaneously in the tail-base with dendritic cells (DCs) pre-loaded with OVA protein immune complexes, containing the specific T cell antigen recognized by OT-I T cells, while control mice received unloaded DCs. Based on previous *in vitro* results, we adopted an *in vivo* imaging protocol with separate administration of the two substrates with a 3 h time interval to allow clearance of the first substrate D-luciferin (as assured by imaging the mouse prior to injection of the second substrate) measuring CBG99 luciferase activity, followed by measurement of PpyRE9 luciferase activity using the CycLuc1 substrate. This order was chosen based on the longer half-life of the second substrate CycLuc1 compared to the first substrate D-luciferin (18). Periodical bioluminescence imaging was then performed, focusing on the vaccine-draining inguinal lymph nodes, and the green and red light signals were quantified. In the first week after vaccination, OVA-vaccinated mice showed a sharp increase in both the constitutive green light signal and the activation-induced red light signal in the lymph nodes, peaking at day 8 after injection (Figure 5A). In contrast, control mice had a comparable constitutive green light signal on day 2 as OVA-vaccinated mice, but did not show a strong T cell expansion and no red light signal above background, suggesting that the DC-OVA vaccine specifically induced activation and expansion of TbiLuc^*^OT-I T cells. Two days after vaccination, T cell activation could be detected by means of PpyRE9 luciferase activity, while T cell expansion as measured by CBG99 luciferase activity started later in time. The dramatic increase in luciferase activity in OVA-vaccinated mice, and the lack of NFAT-dependent red luciferase activity in control mice, can be seen in representative images from day 8 (Figure 5B). To estimate the sensitivity of the TbiLuc mouse model for detecting T cell activation *in vivo*, we repeated the DC-OVA vaccination experiment using lower numbers of DC-OVA cells used as a vaccine, ranging from 10- to 1000-fold lower than the optimal condition of 1 million cells as used in Figure 5, while the number of TbiLuc^*^OT-I T cells remained the same. Imaging of vaccine-draining lymph nodes of mice vaccinated with 10-fold fewer DC-OVA cells showed robust and quantifiable T cell activation, while also a vaccine dose of 100-fold below optimal still produced detectable T cell activation, indicating that the TbiLuc mouse model can detect T cell responses in suboptimal conditions (Supplementary Figure 5). Taken together, these results proof-of-concept for the use of the TbiLuc model to longitudinally visualize T cell responses *in vivo*.

## Discussion

In this study, we report on the design and development of a dual-luciferase T cell transgenic mouse, called TbiLuc, as a novel tool for non-invasive imaging of T cells. The dual-luciferase construct allows live visualization of both the location and the activation status of T cells, which we established in antigen-specific T cell activation studies both *in vitro* and *in vivo*. The TbiLuc mouse model has numerous applications both in fundamental T cell biology and in preclinical translational studies on the many diseases in which T cells play a role. For instance, since it has been established that T cells are crucial in the spontaneous or therapeutically-induced clearance of malignant cells, the TbiLuc mouse can be used to test the efficacy of cancer vaccines, immunomodulatory antibodies and other treatment modalities that depend on T cells. Disease models of viral infection and autoimmunity involving T cell effector cells are other possible applications of the TbiLuc mouse (26, 27).

To introduce luciferase-encoding genes into cells, researchers commonly use retroviral transduction. However, naïve T cells cannot be efficiently transduced unless they are pre-treated by either TCR stimulation with cognate antigen or with cytokines such as IL-7, both of which trigger downstream signaling pathways in the T cells (6–9, 28, 29). This makes studies on truly naïve T cells impossible, although T cell “rested” after activation and transduction are sometimes considered to represent naïve T cells; a perhaps more pragmatic rather than immunological interpretation. Instead, integration of a luciferase-encoding gene into murine oocytes, resulting in a luciferase-transgenic mouse strain, is a time-consuming but scientifically much more attractive alternative that allows the isolation of luciferase-expressing naïve T cells from the lymphoid organs of the mouse. Indeed, several groups have created luciferase reporter mice for T cell imaging and showed the potential of BLI for T cell tracking (10–12). However, these single-luciferase reporter mice only provide information on the location of T cells, without additional information on their activation state. The feasibility of creating dual-luciferase transgenic mice has been shown before, but never using cell type-specific expression (30, 31). We describe the first dual-luciferase T cell transgenic mouse employing D-luciferin analogs, which offers an important advantage in allowing simultaneous visualization of T cell location and activation. For transgenesis, we chose to randomly integrate our construct and select founders for high constitutive green luciferase expression. As the length of the genetic construct (more than 10 kb) influences the efficiency of transgenic production, the inclusion of fluorescent markers in the construct was avoided. We did not use the CRISPR-Cas9 gene knock-in system for transgenesis as it suffers from suboptimal efficiency (1–10%), especially for constructs longer than 2 kb (32).

Luciferase T cell imaging, and optical imaging in general, is especially well-suited for visualization of superficial light signals, as the signal from deeper locations in the body will be influenced by absorption and scattering of photons in the tissues. In this view, radio-imaging provides more accurate information on the location of T cells (33, 34). However, optical imaging strategies avoid the undesired use of radioactive material, and BLI in particular allows the creation of reporter mice where luciferase expression can be restricted to the cell type of choice by choosing a cell-specific promoter. Our choice of the human CD2 minigene as the promoter driving the constitutively expressed CBG99 luciferase is based on earlier studies showing T cell-specific transgene expression (20, 21). The transgenic mouse described by Szyska et al. instead expresses Renilla luciferase under the control of a ubiquitous constitutive promoter, which means that specific T cell tracking is only possible using an experimental setup of T cell isolation and adoptive transfer (13). In the TbiLuc model, the NFAT response element driving PpyRE9 luciferase expression allows a straightforward readout of T cell function, which is valuable extra information besides the location of the T cell. We demonstrated that T cell isolated from TbiLuc and TbiLuc^*^OT-I mice can be used for cell-based assays using a single substrate D-luciferin. On the other hand, an optimized protocol had to be developed for *in vivo* imaging. A limitation of the dual-color system using a single D-luciferin substrate *in vivo* is the fact that red photons detected *in vivo* could be derived from the green luciferase located in deep tissues, based on the superior tissue penetration of high-wavelength (red shifted) photons, as we have described before (17). In addition, NFAT-driven luciferase activity may be observed in non-T cells, as we have shown for NK cells after PMA+ionomycin treatment, however, these cells can be distinguished from T cells because of the absence of green CD2-driven luciferase emission. Moreover, in models using adoptive T cell transfer, NK cells are efficiently excluded during the T cell isolation process. Besides activated T cells, dual-luciferase signals could possibly also be observed during T cell exhaustion, which has been described to be induced by NFAT (35).

Our optimized *in vivo* imaging protocol involves separate injection of the two substrates such that the first substrate is cleared before the second substrate is administered, measuring CBG99 activity using D-luciferin and measuring PpyRE9 activity using CycLuc1. This setup provides the maximal light emission for each luciferase and avoids both biochemical interference from the other substrate and spectral interference from the other luciferase. An *in vivo* vaccination study visualized the expansion and activation of adoptively transferred T cells in vaccine-draining lymph nodes following typical kinetics of T cell responses upon vaccination (36–38). The sensitivity of TbiLuc *in vivo* imaging matched that of a recently reported T cell reporter mouse model, reaching comparable photon fluxes even as we use much a shorter signal acquisition time of 30 s (13). The use of longer acquisition times would likely allow the detection of signals using lower numbers of transferred T cells. A direct quantitative comparison of TbiLuc sensitivity with other bioluminescence reporter systems is complicated by the fact that the sensitivity of a bioluminescence system *in vivo* depends not only on the imaging settings but also on the luciferase-luciferin couple.

Our data have shown that dual-luciferase reporter mice can be easily crossed to other (T cell-) transgenic mouse strains, which brings many possibilities to further fine-tune the reporter system for the biomedical experimental model of interest. Future studies using TbiLuc mice to study T cell dynamics and functionality in diverse experimental (disease) models will help determine the breadth of its applicability. Taken together, this proof-of-concept manuscript introduces the TbiLuc dual-luciferase T cell transgenic mouse that allows to track activation and expansion of T cell populations in naturally organized lymphoid tissue, with full retention of T cell naivety and antigen-specific functionality. Many biomedical research fields can potentially benefit from this advanced live T cell imaging model.

## Author Contributions

JK, LM, FO, and CL designed the transgenic mouse and all experiments. JK and LM performed the experiments, analyzed the data and wrote the manuscript. GZ performed *in vitro* experiments and analyzed the data. MF helped with experimental design and revised the manuscript. JV developed the transgenic mouse. LC and AC advised on and funded the project. FO and CL supervised the project, revised the manuscript and provided funding.

### Conflict of Interest Statement

GZ was employed by Medres, Cologne, Germany. AC was employed by Percuros BV, Enschede, Netherlands. The remaining authors declare that the research was conducted in the absence of any commercial or financial relationships that could be construed as a potential conflict of interest.
