# Supplementary material for: A Dual-Color Bioluminescence Reporter Mouse for Simultaneous in vivo Imaging of T Cell Localization and Function
Source: Front Immunol. 2019 Jan 8;9:3097. doi: 10.3389/fimmu.2018.03097 (PMC6333049; doi:10.3389/fimmu.2018.03097)
Supplement: Supplementary file 1 [file Data_Sheet_1.docx]

Supplementary Material

A dual-color bioluminescence reporter mouse for simultaneous in vivo imaging of T cell localization and function

**Jan Willem Kleinovink^1 *^, Laura Mezzanotte^2,3 *^, Giorgia Zambito^2,3,4^, Marieke F. Fransen^1^, Luis J. Cruz^5^, J. Sjef Verbeek^6^, Alan Chan^7^, Ferry Ossendorp^1 #^ and Clemens Löwik^2,3,8 #^**

^1^ Department of Immunohematology and Blood Transfusion, Tumor Immunology, Leiden University Medical Center, Leiden, Netherlands.
^2^ Department of Radiology and Nuclear Medicine, Optical Molecular Imaging, Erasmus Medical Center, Rotterdam, Netherlands.
^3^ Department of Molecular Genetics, Erasmus Medical Center, Rotterdam, Netherlands.
^4^ Medres, Cologne, Germany.
^5^ Translational Nanobiomaterials and Imaging, Department of Radiology, Leiden University Medical Center Leiden, Netherlands.
^6^ Department of Human Genetics, Leiden University Medical Center, Leiden, Netherlands.
^7^ Percuros B.V., Enschede, Netherlands.
^8^ Department of Oncology, CHUV Lausanne University Hospital, Lausanne, Switzerland.
^*^ These authors have contributed equally to this work.
^#^ These authors share corresponding and senior authorship.

**Correspondence:**Clemens Löwik: c.lowik@erasmusmc.nl
Ferry Ossendorp: f.a.ossendorp@lumc.nl


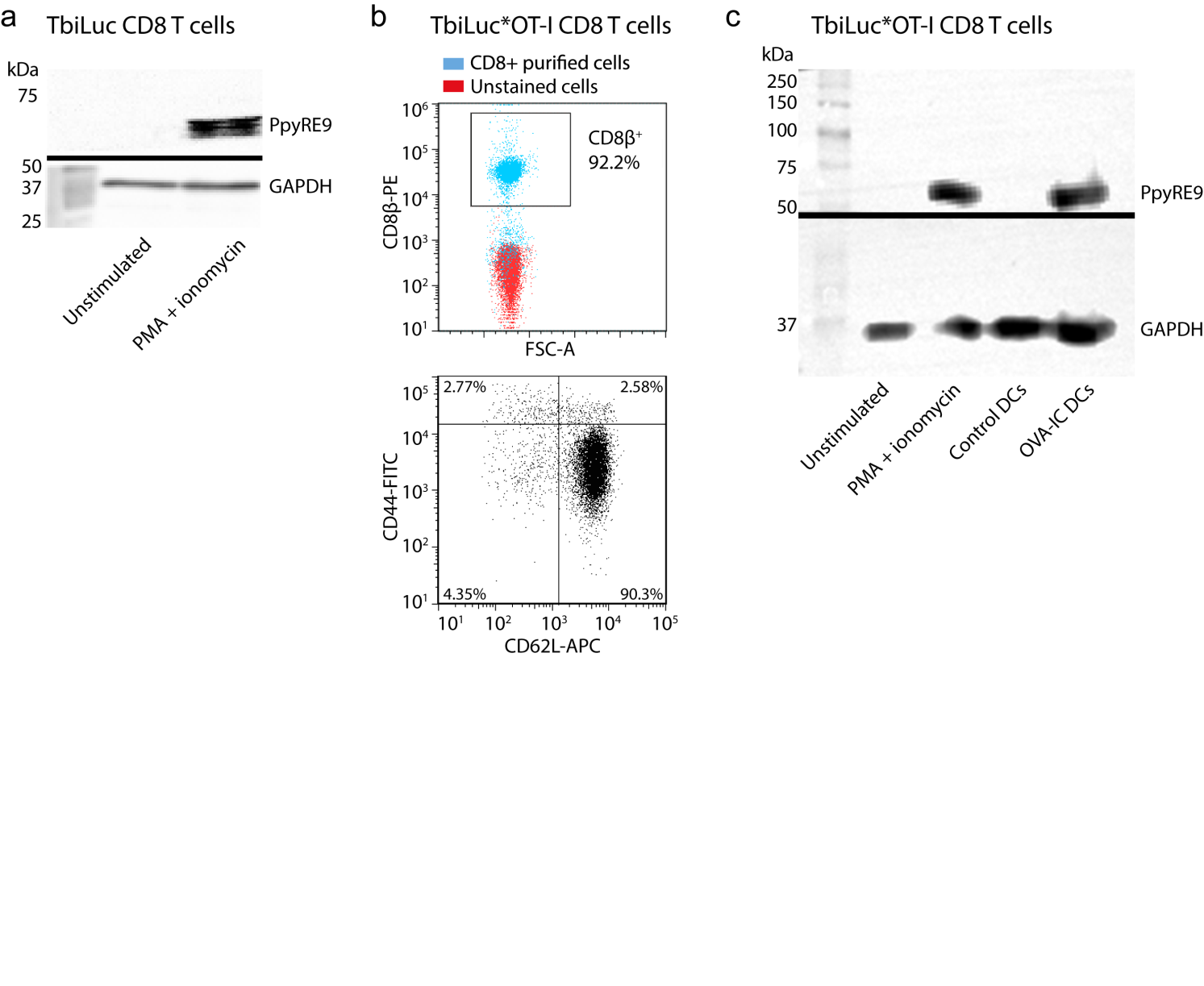


**Supplementary Figure 1. (a)** Western blot for the activation-induced PpyRE9 luciferase. CD8 T cells from TbiLuc mice stimulated with PMA+ionomycin, but not unstimulated cells, produce PpyRE9 luciferase protein. **(b)** Representative flow cytometry plots showing the efficient purification of CD8 T cells (top), which have a naïve phenotype (CD44^lo^, CD62L^hi^, bottom) from TbiLuc*OT-I mice. **(C)** Western blot for the activation-induced PpyRE9 luciferase performed 24 hours after activation. CD8 T cells from TbiLuc*OT-I mice stimulated with PMA+ionomycin or OVA immune complex-loaded dendritic cells (OVA-IC DCs), but not unstimulated cells or control DC-stimulated cells, produce PpyRE9 luciferase protein.

**
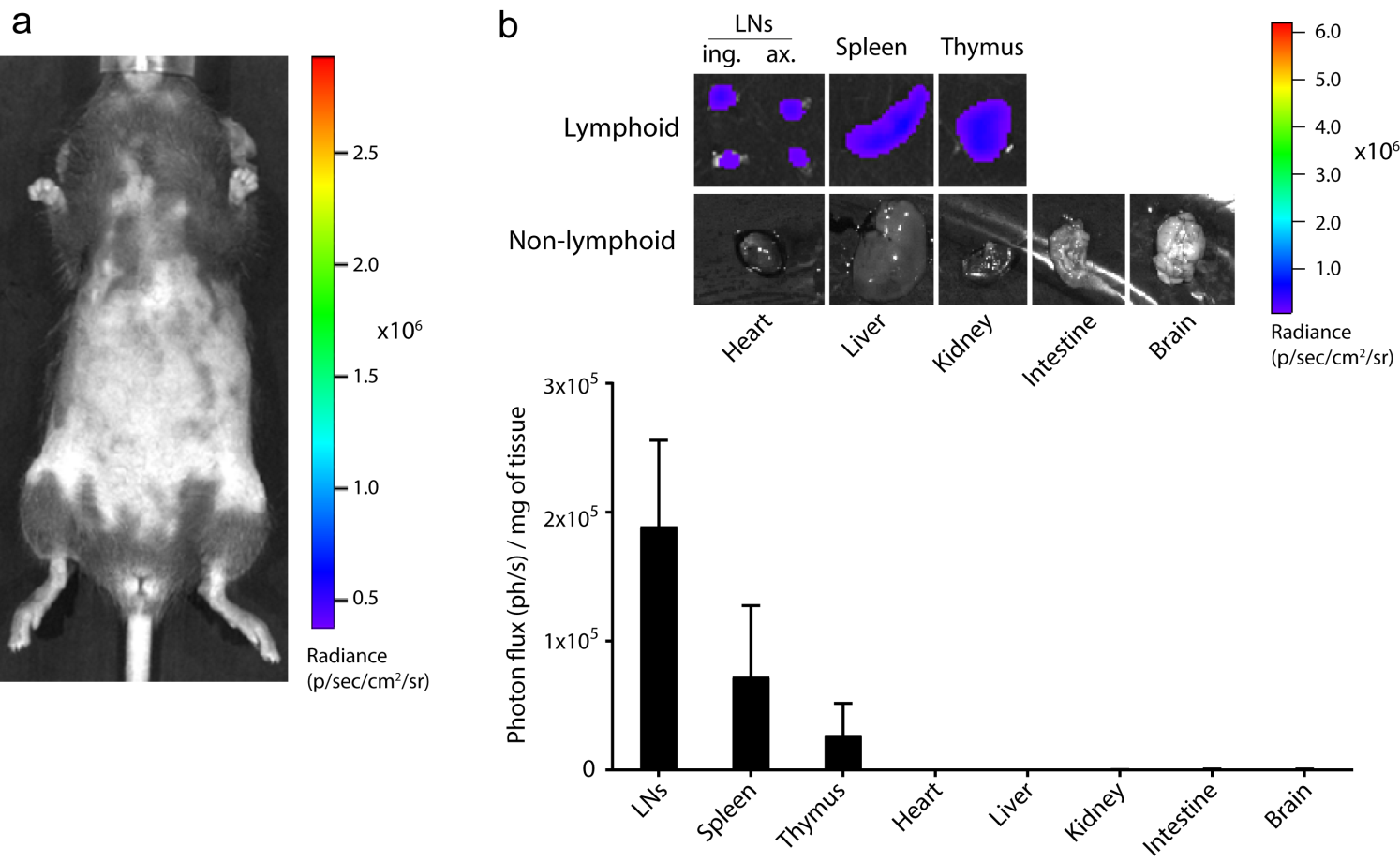
**

**Supplementary Figure 2. (a)** Bioluminescence measurement of a naïve TbiLuc mouse using the CycLuc1 substrate. The abdomen is shaved to reduce signal absorption. **(b)** Representative images and quantification of luciferase signal in several lymphoid and non-lymphoid organs of TbiLuc mice (n=3, mean + SEM). LNs = lymph nodes, ing. = inguinal, ax. = axillary.

**
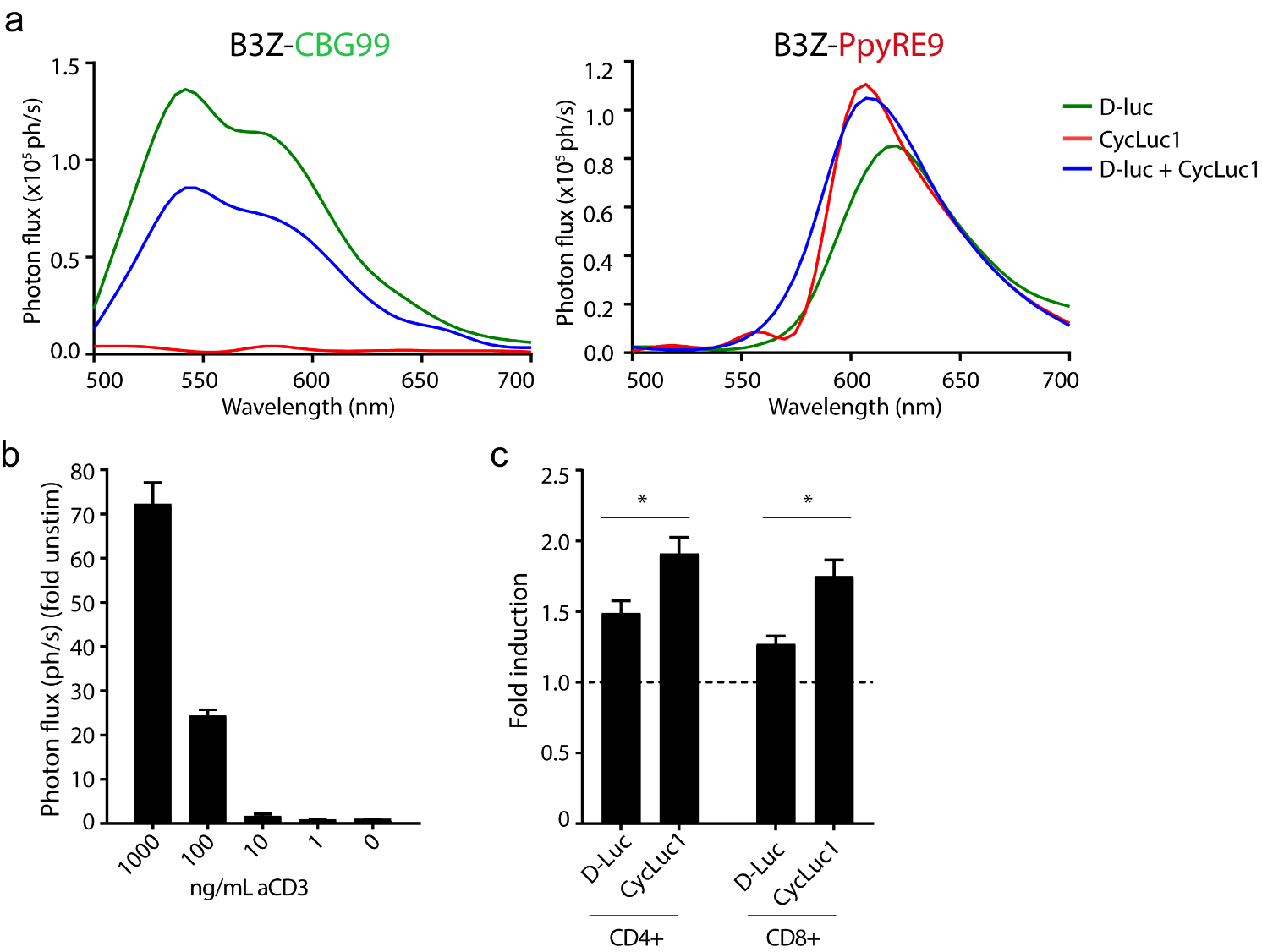
**

**Supplementary Figure S3. (a)** Comparison of the efficacy of D-luciferin (D-luc), CycLuc1, or mixed substrates using B3Z T cell hybridoma cells transduced to constitutively express either green CBG99 (left) or red PpyRE9 (right) luciferases, as indicated. D-luciferin is the optimal substrate for CBG99, and CycLuc1 is the optimal substrate for PpyRE9, but mixing the two substrates reduces signal strength. **(b)** Sensitivity of detection of T cell activation after aCD3 stimulation in vitro. After overnight stimulation of purified TbiLuc*OT-I CD8 T cells with different doses of plate-bound agonistic anti-CD3 antibody, the NFAT-PpyRE9 luciferase signals were measured using the CycLuc1 substrate. Signals were quantified as photons/second, corrected for background are presented as fold-unstimulated (0 ng/mL = 1-fold). Mean + SD of n=3 per condition. **(c)** Fold induction of NFAT-luciferase expression in CD4+ or CD8+ TbiLuc T cells after PMA+ionomycin stimulation compared to unstimulated control cells, measured using either D-luc or CycLuc1 as substrate. Signals are corrected for differences in constitutive hCD2-luciferase expression. Baseline expression (1-fold) is indicated by the dotted line. Statistical significance of differences between D-luc and CycLuc1 data was analyzed by t-test, * = p<0.05.

**
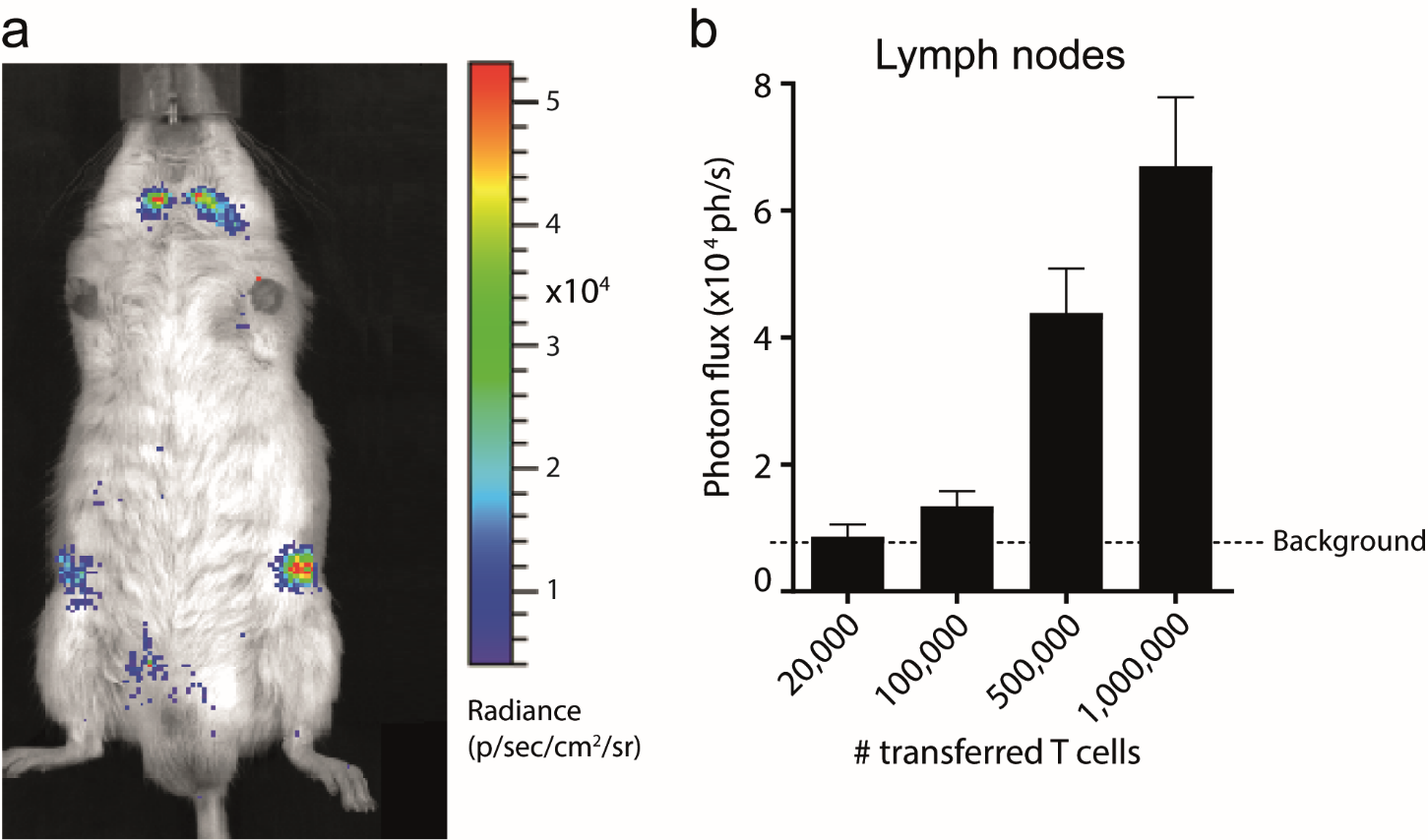
**

**Supplementary Figure S4. Bioluminescence imaging of TbiLuc*OT-I CD8 T cells after adoptive transfer into recipient mice. (a)** Adoptively transferred T cells home to the secondary lymphoid organs of recipient Albino B6 mice. Image acquired using D-luciferin on day 1 after adoptive transfer. **(b)** Quantification of the signals in inguinal lymph nodes showing that signal strength correlates to the number of transferred T cells, n=4 mice per group.


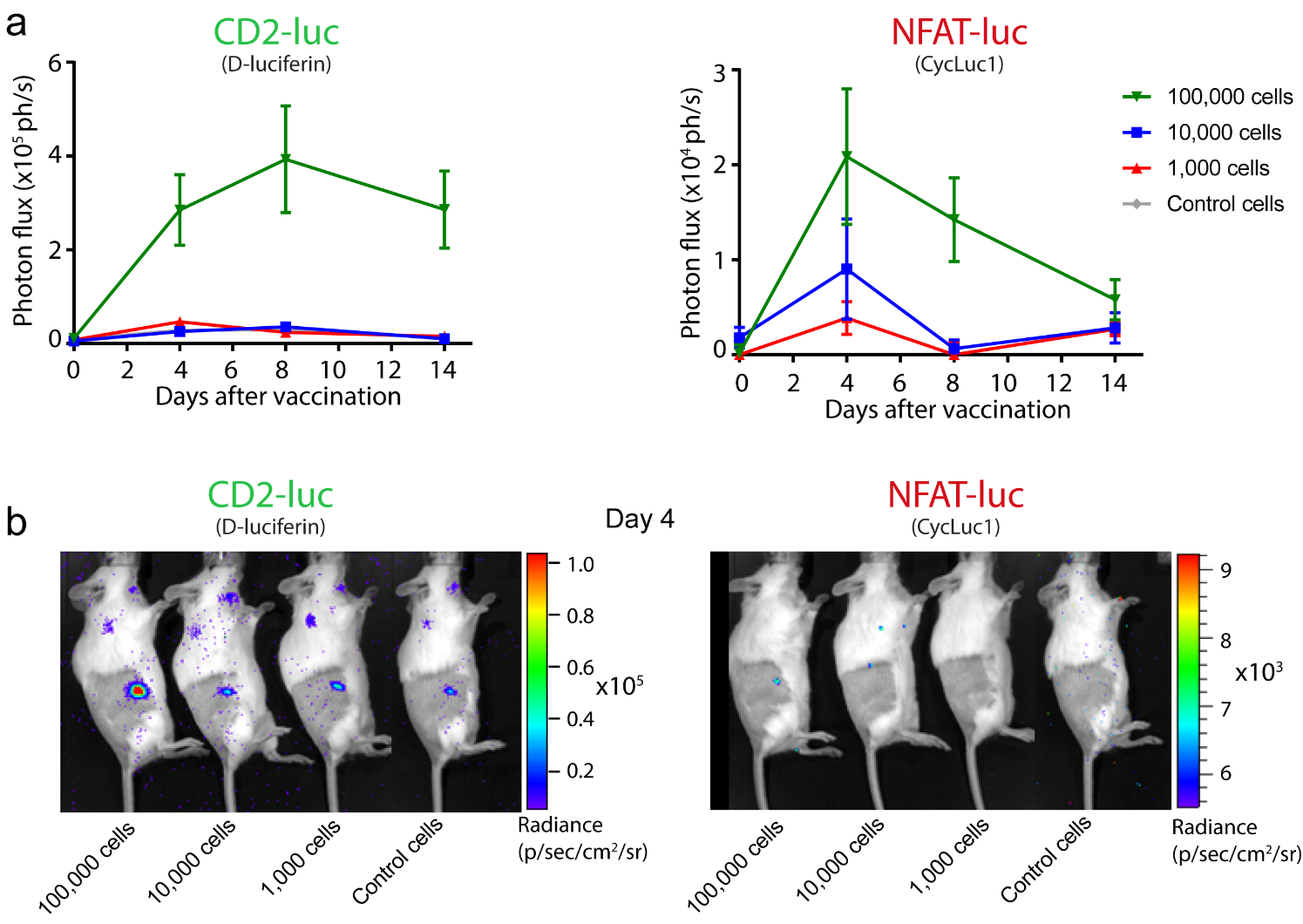


**Supplementary Figure S5. Detection limit of TbiLuc*OT-I CD8 T cells in vivo after vaccination. (a)** Constitutive CD2-luciferase (left) and NFAT-luciferase (right) signals from vaccine-draining inguinal lymph nodes of vaccinated mice. On day 0, mice received adoptive transfer of TbiLuc*OT-I CD8+ T cells and were vaccinated with titrated numbers of OVA immune complex-preloaded dendritic cells or with unloaded control dendritic cells (n=6 per group). CD2-CBG99 and NFAT-PpyRE9 luciferases were visualized using the D-luciferin and CycLuc1 substrates, respectively. Mice receiving the control cells showed no detectable NFAT-luc signal after subtraction of background. **(b)** Representative pictures of constitutive CD2-luciferase (left) and NFAT-luciferase (right) signals in lymph nodes of mice on day 4.

**
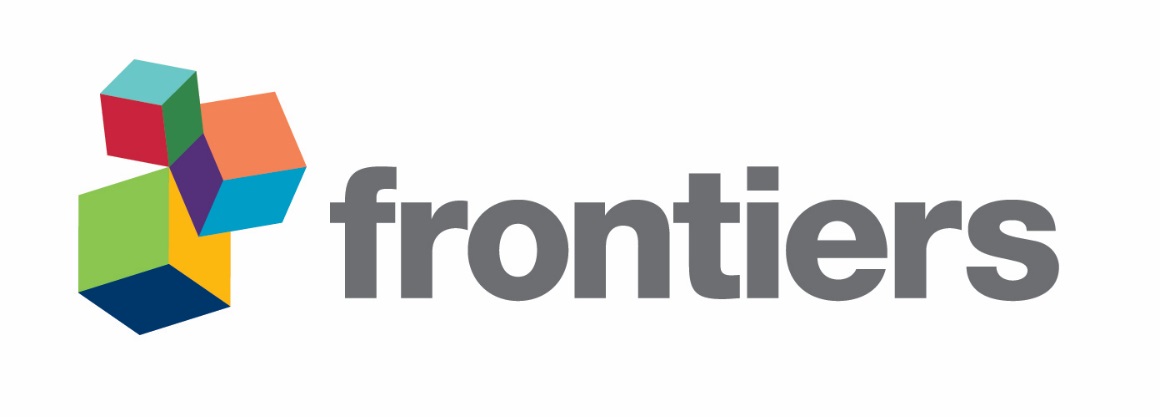
**
